# Preserving Natural Tooth Structure With Biomimetic Restorative Techniques: Current Concepts, Clinical Strategies, and Future Perspectives

**DOI:** 10.7759/cureus.107155

**Published:** 2026-04-16

**Authors:** Mausmee Ved, Niraj Kinariwala, Chintan Joshi, Jahanvi Patel, Dhwani Bhatia, Diya Patel, Sunny Shah, Ishita Gupta

**Affiliations:** 1 Conservative Dentistry and Endodontics, Karnavati School of Dentistry, Gandhinagar, IND; 2 Pedodontics and Preventive Dentistry, Brush and Play Pediatric Dentistry, Cupertino, USA; 3 Dental Surgery, Karnavati School of Dentistry, Gandhinagar, IND

**Keywords:** adhesive dentistry, biologically inspired dentistry, dental restorations, minimally invasive dentistry, tooth remineralization

## Abstract

The scientific study of nature's functional beauty is called biomimetics. The Eiffel Tower's design, which was influenced by the trabecular structure of bone, is one of the most popular examples of using the biomimetics idea. The goals of applying biomimetic ideas and techniques in dentistry are to maintain tooth viability and architecture, enhance the life of restorative dental procedures, and avoid further reduction phases. Biomimetic dental materials are characterized by excellent physical and chemical properties and intrinsic biological compatibility. Because of their improved durability, preservation, revitalization, and antibacterial characteristics, they have been effectively utilized in a variety of dental specialties.

It has also been shown that many biomimetic materials have the potential to overcome the significant shortcomings of their ancestors from earlier generations. This review was conducted to present recent advancements in the developing subject of biomimetics, namely restorative and regenerative dentistry. Many methods for tooth regeneration, remineralization, and restoration are also included in this review. Additionally, a range of biomimetic and tissue engineering dental restorative materials are discussed.

## Introduction and background

With the core goal of using cutting-edge materials and techniques to mimic the complex structure and function of real teeth, biomimetic restorative dentistry represents a significant advancement in dental care [1]. Minimally invasive dentistry prioritizes preservation of sound tooth structure through conservative diagnostic and restorative strategies. Biomimetic dentistry builds upon this concept by seeking to restore teeth in a way that mimics the natural biomechanical behavior of dental tissues. Bioactive materials differ from conventional restoratives in that they actively interact with the surrounding biological environment, promoting remineralization and tissue repair. Although related, these concepts are distinct and collectively contribute to modern tooth-preserving dentistry [1]. Conventional mechanical retention techniques have given opportunity for adhesive and biologically susceptible techniques in restorative dentistry [2]. Modern restorative dentistry aims to preserve teeth's biological vitality and structural integrity in addition to restoring its function and appearance [2,3]. Full-coverage crowns and other traditional restorative procedures frequently require significant removal of healthy tooth tissue, which impairs the tooth's biomechanical qualities and puts it at risk for long-term failures [3].

In order to overcome these difficulties, the biomimetic approach of dentistry was developed, which replicates the tooth's intrinsic biomechanical, aesthetic, and functional properties [3]. Utilizing cutting-edge adhesive innovations, reinforcement of fibers, biologically active materials, and regeneration protocols, biomimetic restorative dentistry replicates enamel and dentin to increase lifespan and maintain pulp vitality [4].

Molar-incisor hypomineralization represents a clinically challenging scenario where biomimetic and minimally invasive restorative strategies are particularly valuable [4]. Enamel affected by molar-incisor hypomineralization is porous, hypersensitive, and prone to post-eruptive breakdown, often complicating conventional restorative approaches. Adhesive restorations, bioactive and remineralizing materials, and resin infiltration techniques align well with biomimetic principles by preserving residual tooth structure while reinforcing compromised enamel. Recent literature highlights the importance of conservative and biologically oriented management strategies in molar-incisor hypomineralization to improve long-term outcomes and patient comfort [5].

Therefore, biomimetic restorative dentistry represents a comprehensive clinical philosophy that integrates biological principles, material science, and adhesive technology to preserve natural tooth structure while restoring optimal function and esthetics [5,6]. This narrative review aims to explore current concepts, clinical strategies, supporting evidence, and future perspectives in biomimetic restorative techniques, highlighting their role in advancing conservative and biologically driven dental care. The aim of this review paper is to offer a clear elucidation of its scope, various domains of biomimetic dentistry, and the materials utilized in biomimetics that enhance tooth strength.

## Review

Methodology

This review carefully analyzed literature on biomimetic principles and advancements in restorative dentistry using online resources such as PubMed, Google Scholar, Web of Science, and EBSCOhost. "Biomimetic dentistry", "biomimetic restorative techniques", "bioactive restorative materials", "tooth preservation", "immediate dentin sealing", and "deep margin elevation" were among the search terms used. The last search was performed in January 2026, and studies published between 2000 and 2025 were considered. Peer-reviewed publications, review papers, and appropriate books were taken into account for inclusion. Articles were included if they were peer-reviewed clinical studies, in vitro studies, systematic reviews, or narrative reviews relevant to biomimetic restorative concepts. Non-English publications, conference abstracts, and studies not directly related to restorative dentistry were excluded. Priority was given to recent high-quality evidence, including randomized controlled trials, systematic reviews, and clinically relevant investigations. After first screening titles and abstracts for relevancy, studies that might be eligible were evaluated in full text. The last collection of included literature offers a thorough examination of the most recent data on the use of biomimetic concepts, bioactive materials, and cutting-edge restoration methods meant to maintain the integrity of natural teeth.

Biomimetic restorative dentistry

Restoring the hard tissues (cementum, dentin, and enamel) is the primary goal of biomimetic restorative dentistry. To achieve optimal functional outcomes, the restorative material should be capable of closely replicating the natural tooth’s biomechanical properties. This gives the tooth nearly normal biology and appearance and enables it to act as a single unit against functional stresses [5]. A comprehensive comparison of biomimetic and conventional restorative dentistry is given in Table 1, which also emphasizes the key distinctions between the two approaches across a number of criteria. This contrast is necessary to comprehend the biomimetic's distinct value proposition, strategy, and how it differs from traditional techniques [6].

**Table 1:** Comparison of biomimetic and conventional restorative dentistry The table is created by Mausmee Ved, the author of this article

Criterion	Biomimetic Restorative Dentistry	Traditional Restorative Dentistry
Philosophy	Seeks to replicate the natural form, function, and biomechanics of teeth	Primarily addresses dental disease through mechanical repair and replacement
Primary goal	Conservation of natural tooth structure and long-term functional preservation	Management of existing pathology, often by replacing or removing compromised tissues
Approach to tooth structure	Emphasizes minimally invasive techniques, preserving up to 70–90% more healthy tissue	Frequently involves removal of sound tooth tissue to accommodate restorative procedures
Materials utilized	Advanced composites, ceramic inlays/onlays, bioactive cements, and emerging plant-based materials	Conventional amalgams, porcelain-fused-to-metal crowns, and traditional ceramic systems
Level of invasiveness	Minimally invasive; avoids unnecessary drilling, grinding, or extensive preparation	More invasive; extensive tooth preparation and removal of healthy tissues are common
Esthetic outcome	Provides superior esthetics with tooth-colored, biomimetic materials blending seamlessly	Esthetics may be limited, particularly with metal-containing or opaque materials
Durability	Demonstrates long-term success (≥90% survival over 10 years) with improved sealing ability	Restorations may weaken the remaining tooth and often require more frequent replacement
Post-operative complications	Reduced risk of pulp irritation, hypersensitivity, recurrent caries, or fracture	Increased likelihood of sensitivity, pulpal damage, and structural failure
Need for endodontic or crown therapy	Conservative nature lowers the necessity for root canal treatment or full-coverage crowns	Aggressive preparation often leads to higher incidence of endodontic therapy and crowns

Biomimetic integration of adhesive reinforcement and functional stress management

The basic biomimetic restorative dentistry ideas may be separated into two categories by the authors: bond-maximizing procedures and stress-reducing protocols (Table 2) [7].

**Table 2:** Biomimetic integration of adhesive reinforcement and functional stress management This table is created by Mausmee Ved, the author of this article. It summarizes and synthesizes data extracted from the published literature [7]. The content has been substantially modified and reformatted by the author.

Approaches to Strengthen Adhesive Integrity	Methods to Minimize Structural and Functional Stress
Develop a continuous, caries-free enamel perimeter to ensure an uncompromised marginal seal	Replace compromised occlusal and proximal enamel with indirect or semi-direct restorations to improve load distribution
Perform controlled micro-etching or air-particle abrasion to increase surface roughness and surface energy	Adopt staged restorative procedures to permit polymerization stress relaxation over time
Extend preparation margins with enamel beveling to increase bonding substrate area and improve esthetic blending	Reinforce restorations by embedding fibers within axial walls and pulpal floors to enhance fracture resistance
Utilize advanced, evidence-based adhesive systems with superior long-term bond stability	Preserve cuspal integrity by maintaining cusp thickness below 2 mm in onlay preparations
Implement immediate dentin sealing to protect freshly cut dentin and improve hybrid layer quality	Modify occlusal morphology to channel forces along the long axis of the tooth
Elevate deep cervical margins to a supragingival level to facilitate optimal bonding and isolation	Design restorations that favor vertical force transmission rather than lateral stress concentration

Creating a peripheral seal zone free of dental caries

In order to guarantee restoration strength and endurance, biomimetic restorative dentistry concentrates on striking the perfect balance during meticulous dentin removal [8]. It highlights how crucial it is to establish a peripheral seal zone that includes the dentin-enamel junction, caries-free enamel, and superficial dentin for the best possible connection [9]. Due to the limitations of caries-detecting dyes, minimally invasive dentistry advises the visual-tactile approach, while biomimetic restorative dentistry recommends using caries-detecting dyes to diagnose and guide the removal of carious tissue. This idea was first proposed by Fusayama in the 1970s [9]. At first, it was thought that using a 0.5% basic fuchsin-propylene glycol solution would be a less subjective method of differentiating the layers of carious dentin [10].

Aluminum oxide air abrasion

Abrasive particles driven by compressed air are used in air abrasion, a process that dates back to the 1940s and modifies material surfaces. Since then, a number of tools have been created for uses such as surface roughening or polishing, staining, cavity preparation, prophylaxis, selective caries removal, and tribochemical coating [11]. Al2O3 air abrasion is recommended for cleaning cavity surfaces, eliminating residues, and improving bonding by producing a rough surface for micromechanical retention [12].

Bevel enamel in posterior restorations

Because enamel prisms stand at a straight angle to the cavosurface in proximal boxes, biomimetic restorative dentistry advises putting a 45° bevel there. To increase bonding and marginal adaption, eliminate any compromised enamel, and improve the composite resin's aesthetic blending, the cavosurface angle in occlusal margins should be flattened with a tiny bevel using a fine diamond bur [13].

Use of the best adhesives available

Clinicians can use either the self-etch method or the etch-and-rinse technique while carrying out adhesive restorative procedures. Many researchers and doctors have regarded the adhesive brands OptiBond FL (Kerr Corp; Orange, CA, USA) and Clearfil SE Bond (Kuraray; Tokyo, Japan) as the "gold standard" materials among the three-step etch-and-rinse and two-step self-etch adhesives [14].

Immediate dentin sealing and resin coating

In order to maintain pulp vitality and seal open tubules and prevent water seepage, the process depends on shielding freshly cut dentin in indirect preparations from the dentin [15]. Dentin adhesives must be applied before the imprint or provisional phase of indirect restorations, occasionally with an extra flowable composite layer on top [16].

Deep margin elevation

Subgingival margins in class II cavities provide significant challenges because of limited access and difficulties in maintaining isolation from blood, saliva, and crevice fluid [17]. To move the cervical margin coronally, a base of composite resin is applied over the current cervical margin [18]. This method was comparable to the open-sandwich technique, which fills the cervical portion of the proximal box with resin-modified glass ionomer cements to enhance dentin bonding and lessen polymerization shrinkage pressures [19].

Fiber embedded in the axial walls and pulpal floor

Although replacing badly degraded teeth can be challenging, several approaches have been put forth, such as crowns, cuspal coverage amalgam, endodontic therapy with glass fiber post-cementation, direct composite restorations, and composite/ceramic onlays [20]. For the pulpal wall and surrounding cavities, biomimetic restorative dentistry recommends fiber-reinforced composites made of polyethylene fibers such as EverX (GC Dental, Tokyo, Japan) and Ribbond (Ribbond, Inc., Seattle, Washington, USA) [21]. Walls in big posterior composite restorations to lessen the stress caused by polymerization shrinkage [22]. According to this approach, gap development is decreased and fracture resistance is increased [23]. While it also asserts that this process can strengthen bonds, this is not the case when contrasted with the conventional composite stacking method [24].

Current research and future trends in biomimetic restorative dentistry

As researchers and clinicians seek to enhance restorative outcomes by maintaining natural tooth structure and function, biomimetic restorative dentistry has drawn a lot of interest recently. In order to replicate the biomechanical characteristics of natural teeth, current research highlights the significance of adhesive systems, bioactive restorative materials, and minimally invasive approaches [25]. Recent developments in bioactive materials, including resin composites with remineralizing fillers, bioactive glass, and cements based on calcium silicate, have demonstrated encouraging outcomes in enhancing dentin remineralization and lowering microleakage [26]. These materials are better than conventional inert restorative materials because they actively aid in the repair of oral tissues in addition to restoring function [27].

Another emerging field in biomimetic dentistry is nanotechnology. When added to composites and adhesives, nanoparticles enhance their mechanical characteristics, antibacterial capability, and remineralization ability [28]. One important avenue for the future is the development of smart restorative materials that react to pH changes and release ions in response to cariogenic challenges [28,29].

The integration of biomimetic and bioactive materials has significantly expanded the scope of regenerative dentistry. Unlike conventional restorative materials that function primarily as passive replacements, bioactive materials actively interact with the surrounding biological environment to promote repair and regeneration. The controlled release of calcium and hydroxyl ions promotes alkaline pH conditions favorable for hard tissue formation and antibacterial action [28,29]. Additionally, materials capable of forming hydroxyapatite-like structures at the interface improve sealing ability while biologically integrating with dentin. Calcium silicate-based cements, bioactive glass, ion-releasing restorative systems, and advanced adhesive materials have demonstrated the ability to stimulate mineral deposition and enhance dentin-pulp complex healing [29].

With the addition of tissue engineering, stem cell treatments, and bio-inspired scaffolds, it is anticipated that regenerative and biomimetic integration will grow in the future. Through the creation of restorations with extreme precision and natural biomimicry, digital technologies such as computer-aided design and computer-aided manufacturing and 3D printing will further enhance biomimetic principles [30].

Challenges

Although biomimetic restorative dentistry has demonstrated encouraging results, a number of obstacles stand in the way of its complete incorporation into routine practice:

Technique sensitivity: advanced clinical skill is needed for biomimetic operations such as deep margin elevation and rapid dentin sealing. Results can be jeopardized by even small mistakes in bonding or isolation [31,32].

Material constraints: although bioactive and nanotechnology-based composites have advanced, problems like wear, polymerization shrinkage, and adhesive compatibility persist [33].

Cost and accessibility: in many contexts, the use of computer-aided design and computer-aided manufacturing, bioactive ceramics, and smart composites restricts access and raises treatment costs [34].

Absence of defined procedures: disparities in restorative decisions, bonding tactics, and layering methods lead to inconsistent therapeutic outcomes [35].

Long-term proof: large-scale randomized controlled studies with prolonged follow-ups are still required, despite the encouraging short-term evidence [36,37].

Incorporation of conservative dentistry into biomimetic materials

The material selected to restore the tooth's functionality should have qualities such as modulus of elasticity, tensile strength, and compressive strength for the repaired tooth structure (Table 3) [38].

**Table 3:** Biomimetic materials in conservative dentistry The table is created by Mausmee Ved, the author of this article Source: Modified from Paryani et al. [39] Ca(OH)₂, calcium hydroxide; PUF, polyurea-formaldehyde; Ca, calcium ion; Ca-P, calcium-phosphate ion

Material	Composition/Key Feature	Properties	Uses in Dentistry	Notes/Limitations
Hydroxyapatite	Calcium phosphate, similar to bone	Osteoconductive, poor mechanical strength	Bone grafting, filler in composites, perforation repair, apical barrier formation, pulp capping	Not used in load-bearing areas
Glass ionomer cement	Fluoro-aluminosilicate glass + polyacid	Adhesive to tooth, fluoride release, thermal expansion like dentin	Bonding, lining, luting, sealing, restorations	Moderate strength, moisture sensitive
Calcium hydroxide	Ca(OH)₂	Alkaline pH, antibacterial, induces mineralization	Cavity liner, pulp capping, root canal dressing/sealer, root resorption treatment	High solubility, weaker sealing
Self-healing composites	PUF or silica microcapsules	Release resin when cracked → polymerizes to repair	Experimental restorative material to improve longevity	Still under research, not routine clinical use
Mineral trioxide aggregate	Calcium silicates, aluminates, gypsum	Hydrophilic, bioactive, induces hydroxyapatite & dentin bridge	Pulp capping, apexogenesis, apexification, root-end filling, perforation repair	Long setting time, costly, handling issues
Ceramir	Calcium aluminate-based	Bioactive, reacts with phosphate to form hydroxyapatite, good gingival response	Luting agent for crowns, inlays	Mostly for cementation
TheraCal	Light-cured, resin-modified silicate	High calcium release, low solubility, protective liner	Pulp capping, liner under composite/amalgam	Resin component may shrink
Bioactive glass	Silica-based glass	Biocompatible, forms Ca-P layer in fluids	Implant coating, bone graft, dentin hypersensitivity treatment	Limited mechanical strength
Emdogain	Enamel matrix derivative	Induces periodontal tissue regeneration	Vital pulp therapy, pulpotomy, replantation (reduce resorption)	Biologic material, expensive
Ceramicrete	Calcium-based, cerium oxide, hydroxyapatite	Releases Ca and phosphate, high sealing ability, biocompatible	Root-end filling, regenerative applications	Long setting time (2.5 h), initial low pH

Hydroxyapatite

Hydroxyapatite is one calcium phosphate material that cannot be replenished. It resembles bone and has an osteoconductive quality. It is used as a filler in composite resin and in bone grafting [38]. In endodontic therapy, hydroxyapatite has been used for pulp capping, apical barrier creation, perforation repair, and periapical defect repair. Malik et al. claim that compared to reparative dentin created using calcium hydroxide, tricalcium phosphate hydroxyapatite produces dentin mineralization that is thicker and more extensive [40].

Glass ionomer cement

Because glass ionomer cement has properties similar to dentin, including fluoride release and adherence to tooth surfaces, it is considered a biomimetic material [41]. Bonding, lining, luting, sealing, and tooth restoration are among the few different uses of this bioactive restorative material. Its coefficient of thermal expansion is identical to that of a naturally occurring tooth [42].

Calcium hydroxide

Hermann was the first to introduce calcium hydroxide in dentistry. It dissociates into calcium and hydroxyl ions, which contribute to its antibacterial properties and stimulate hard tissue formation. Calcium-dependent pyrophosphatase improves as an outcome, which lowers inhibitory pyrophosphate levels and promotes mineralization [42]. It can be used as a temporary root canal dressing to promote the development of hard tissue, a permanent root canal sealer, a cavity liner, or a remedy for root fracture and resorption. Because of its alkaline pH, it has antibacterial qualities and can help dissolve bacteria, their metabolites, and necrotic tissue remnants. Additionally, it may encourage the development of tertiary dentin [43].

Self-healing composites resin

Polyurea-formaldehyde and silica microcapsules are examples of self-healing composites. A medicinal substance, such as water or polyacid, is used in silica microcapsules. When composite resin exhibits visible fractures, the surrounding microcapsules are destroyed and the resin is released. The resin polymerizes, and the crack is sealed when the catalyst added to the epoxy composite reacts with the resin to fill it [44].

Mineral trioxide aggregate

In 1990, Torabinejad developed mineral trioxide aggregate, a hydrophilic material composed of calcium silicate. When it comes into contact with liquids containing phosphate, it crystallizes calcium hydroxide and creates crystals that resemble hydroxyapatite. Its pH ranges from 10 to 12. Mineral trioxide aggregate is composed of gypsum, tricalcium aluminate, dicalcium silicate, tetra-calcium alumino-ferrite, and tricalcium silicate [44].

This is the material of choice for pulp capping, apexogenesis, apexification, vital pulp therapy, and root-end filling in apicoectomy procedures. Following transplantation, odontoblast-like cells proliferate and develop into a collagen matrix. It is less soluble than calcium hydroxide and does not cause tunnel defects when used in vital pulp therapy [45].

Ceramir

Ceramir, which contains calcium aluminate, is used for long-term cementation. All zirconia, inlay, gold, and fixed partial dentures have their dentin and enamel restored by the reaction of the released calcium with the alkaline pH. When used as a luting agent, its superior gingival reactivity and advantageous interaction with the inorganic phosphate in saliva enable it to produce hydroxyapatite [45].

Bioactive glass

Bioactive glass can react with water and other liquids. Because of their properties and outstanding biological compatibility, silica gel polycondensed coatings over glass bulk are likely to be used as precursors for the synthesis of calcium phosphate [46]. Biological regeneration has further advanced with the use of bioactive glasses, which promote tissue repair and regeneration. The procedures of bone augmentation, implant-related coatings, and hypersensitivity of dentin can all benefit from these [47].

Emdogain

Emdogain is composed of propylene glycol alginate as a matrix and enamel matrix protein from swine tooth germs [46]. Emodin also contains non-collagen proteins such as tuftelin, growth factor, enamelin, ameloblastin, and bone morphogenic protein. Because it promotes the development of reparative dentin, it has been used for pulpotomy and vital pulp therapy. During replantation procedures, it is used to reduce the risk of external root resorption [46].

Ceramicrete

Ceramicrete, a novel calcium-based radiopaque filler, is composed of powdered hydroxyapatite and cerium oxide [48]. It is radiopaque and biodegradable and produces calcium and phosphate ions when settled. It has a higher sealing ability than Pro-Root MTA when used as a roots-end filler material. When the Ceramicrete material is submerged in a phosphate-containing fluid, its surface produces hydroxyapatite or dicalcium phosphate dihydrate. The setting-up time is 2.5 hours. Its pH rises with time from its starting point of 2.2 [49].

## Conclusions

In dental care, biomimetic restorative dentistry signifies a dramatic paradigm shift, eschewing conventional mechanical procedures in favor of the idea of "mimicking life." In this method, priority is given to maximum preservation of the natural tooth structure, careful replication of its visual and biomechanical qualities, and the proactive preservation of the pulp. The evolution of adhesive dentistry, from its fundamental principles to a comprehensive understanding of biomechanics, reflects the increasing complexity of clinical practice and underscores the critical interplay between advanced material science and precise clinical techniques.

The biological regeneration of complete tooth structures is the ultimate goal of biomimetic restorative dentistry, providing really regenerative and harmonious treatments that complement the body's inherent procedures, indicating a more conservative, predictable, and biologically integrated dental future.
